# Eruptive Pseudoangiomatosis in Adults: A Case Report of an Unfamiliar Association With Neurological Symptoms

**DOI:** 10.7759/cureus.75747

**Published:** 2024-12-15

**Authors:** Waseem Alhawsawi, Basant A Alzubaidy, Omnia Atif Sulimani, Houriah Y Nukaly, Dhuha O Alquhra Alotaibi, Abdullah M Alharthi, Wesam S Alharbi, Noor M Alharbi, Khalid Al Hawsawi

**Affiliations:** 1 Dermatology, King Fahad Armed Forces Hospital, Jeddah, SAU; 2 Medicine, Umm Al-Qura University, Makkah, SAU; 3 Medicine and Surgery, Batterjee Medical College, Jeddah, SAU; 4 Medicine and Surgery, Umm Al-Qura University, Makkah, SAU; 5 Dermatology, King Abdulaziz Hospital, Makkah, SAU; 6 Internal Medicine, Umm Al-Qura University, Makkah, SAU

**Keywords:** adult, case report, eruptive pseudoangiomatosis, neurology, syncope

## Abstract

Eruptive pseudoangiomatosis (EP) is a rare cutaneous condition that usually resolves spontaneously within a few days and is more frequently seen in the pediatric age group. It is characterized by the sudden onset of asymptomatic small erythematous hemangioma-like papules encircled by a pale halo. The precise pathogenesis is unknown; however, multiple environmental triggers have been reported. This case report aims to highlight the complex presentation of EP with concurrent neurological symptoms in an adult patient.

Herein, we report a 26-year-old male, not known to have any medical illnesses, admitted to our hospital as a case of a motor vehicle accident due to a syncopal attack while driving. Multiple previous syncopal attacks passed unnoticeably in the previous couple of months without being investigated. During admission, dermatology was consulted for an abrupt onset of small discrete asymptomatic, bright red well-delimited macules and papules, measuring 2-3 mm in diameter, surrounded by a pale halo located on the distal upper and lower extremities with the involvement of palms and soles. Neurological evaluation revealed bilateral lower limb weakness and sensory deficits. A skin biopsy was non-specific due to delayed sampling. Syphilis serologies and other labs were unremarkable. The patient has been investigated by neurology and is currently on an implantable loop recorder.

This case underscores the importance of considering EP in the differential diagnosis of abrupt dermatological eruptions, particularly when accompanied by neurological symptoms, warranting further investigation into potential underlying conditions.

## Introduction

Eruptive pseudoangiomatosis (EP) is a rare and benign disease initially identified by Cherry et al. in 1969 at the pediatric age. This disease is characterized by acute eruptive angioma-like papules surrounded by a perilesional pale halo, small in diameter, and resolve spontaneously [1]. The specific etiopathogenesis of EP is unknown; however, multiple triggers have been reported in the literature, including viral infections such as parvovirus B19 and SARS-CoV-2, mosquito bites, flea bites, certain diets, medications, and COVID-19 vaccination. Familial factors may also play a role, as well as immunocompromising disorders, which are closely related to adult individuals [1-6]. EP can affect both pediatrics and, to a much lower extent, adults on different sites of the body and could be associated with prodromal symptoms, including constitutional, upper respiratory tract, and/or GI symptoms [1-3]. This case report presents a complex case of EP in a young adult male patient who concurrently experienced significant neurological symptoms, prompting a thorough clinical evaluation. While primarily a dermatological condition, the association of EP with neurological symptoms has not been documented in the literature and possibly warrants further detailed assessment. Interestingly, EP is more commonly reported in pediatric populations, making this adult presentation notable.

## Case presentation

**Figure 1:**
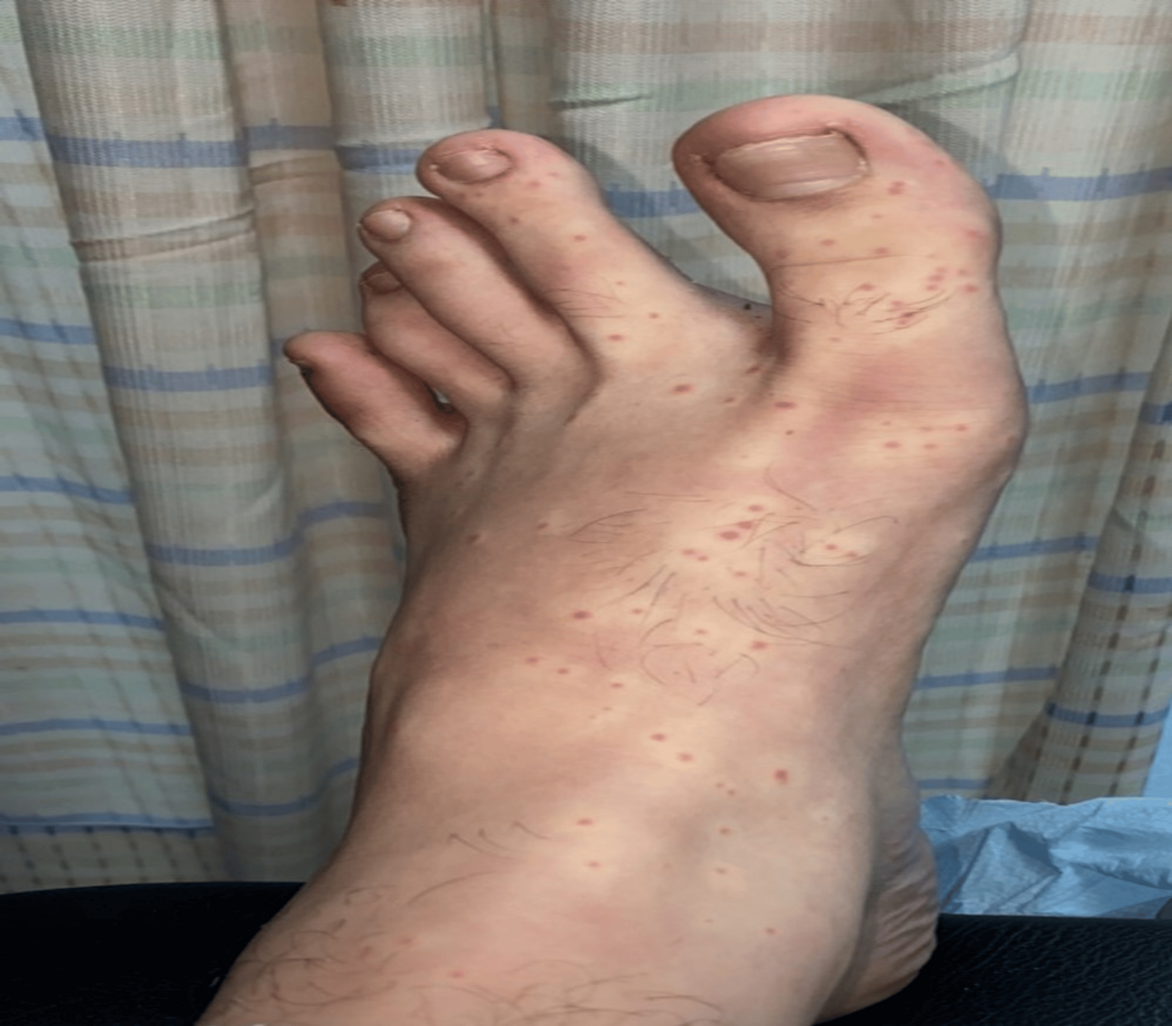
Bright red papules over pale background on the feet dorsa

**Figure 2:**
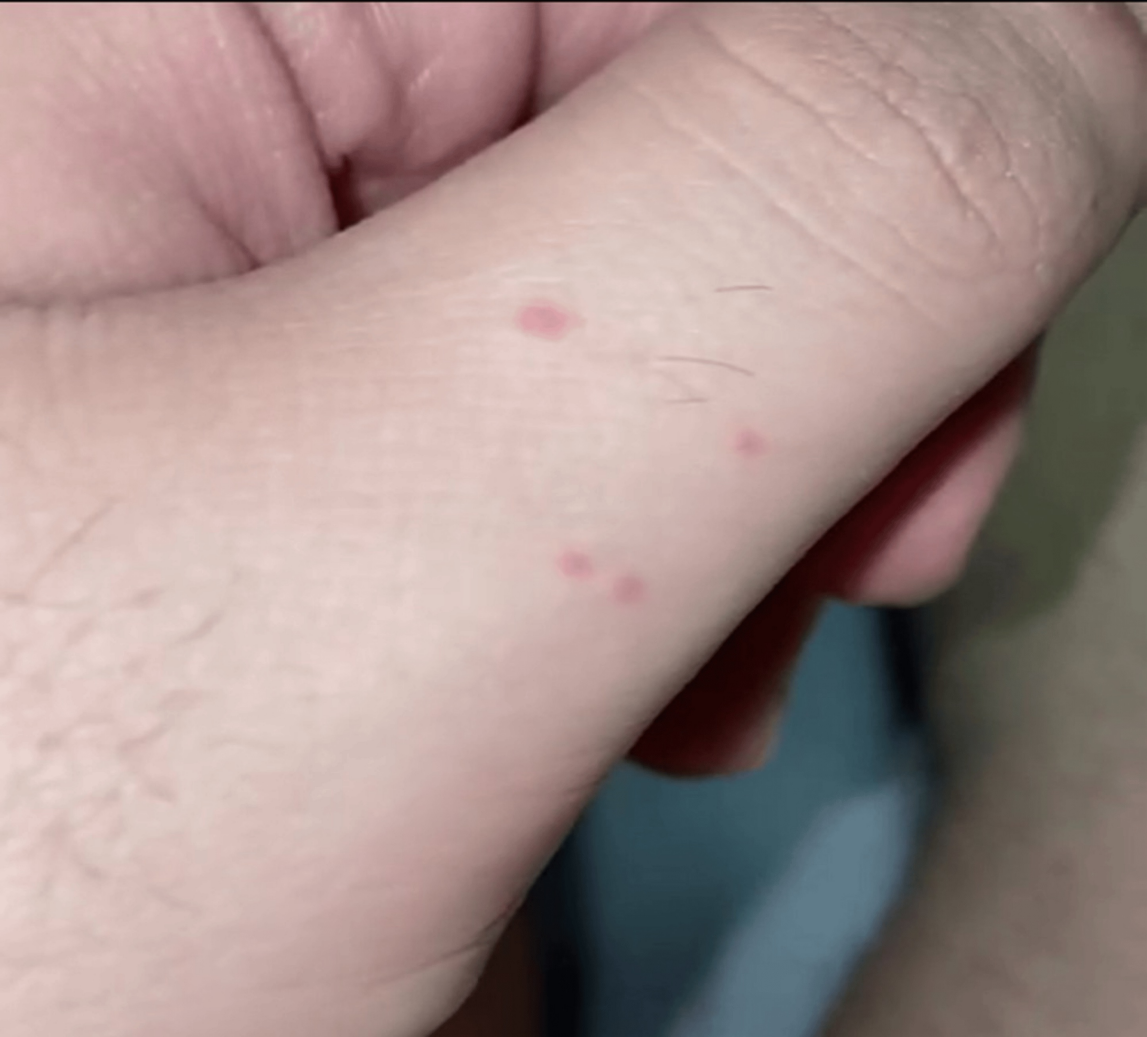
Bright red non-scaly blanching papules over the thumb dorsum

A 26-year-old male with a history of syncopal attack three months ago presented with an abrupt onset of asymptomatic small but numerous skin lesions on his distal extremities, particularly on the palms and soles, for three weeks. The skin rash started suddenly without systemic complaints, and there was no history of recent common infections such as upper respiratory tract infections. Lesions intensity peaked over three weeks and then regressed and healed over another three weeks. No similar rash was noticed previously. He was assessed during admission due to a syncopal attack while driving his car, which led to a motor vehicle accident. During admission, the patient reported vertigo, urinary incontinence, lower limb numbness, and difficulty walking. Neurological examination revealed bilateral lower limb weakness 4/5 and 3/5 for both right and left knee extension, respectively. No signs of dysarthria, nystagmus, ataxia of the extremities and trunk, or muscle stretch reflex abnormalities. Dermatological examination identified discrete 2-3 mm bright red maculopapular lesions with pale halos over the distal upper and lower extremities (Figures 1-2).

A skin biopsy was performed, although histopathological findings were nonspecific due to delayed sampling (approximately six weeks after rash onset), as our patient was hesitant to undergo a skin punch biopsy. The biopsy showed mild hyperkeratosis only. Syphilis serologies were negative, and other laboratory tests, including a complete blood count, metabolic panel, and inflammatory markers, were unremarkable. Given the clinical presentation and dermatological findings, a diagnosis of EP was made. Brain CT demonstrated a well-defined extra-axial cystic lesion with a CSF density at the posterior fossa's right side, suggesting an arachnoid cyst. Spinal skeletal MRI showed multiple disc-osseous complexes with variable degrees of nerve root compression and multilevel central canal narrowing evident at T7-8. Neurology and cardiology consultations were made for further investigations. A cerebral angiogram showed basilar and posterior cerebral artery atherosclerotic changes. He was discharged with an implantable loop recorder. The patient was managed supportively for his dermatological condition, as EP is typically self-limiting.

## Discussion

EP is a rare condition that presents with sudden onset erythematous papules, often linked to viral infections or insect bites. The lesions, characterized by their bright red appearance with pale halos, typically resolve spontaneously. The pathogenesis of EP involves transient vasodilation and endothelial cell swelling, although the exact mechanism remains unclear [3-5]. In this case, the concurrent presentation of neurological symptoms, including vertigo and bilateral lower limb weakness, is particularly notable. While EP primarily affects the skin, this case suggests a potential link between dermatological and neurological manifestations. Brain and spinal imaging findings may explain the patient's neurological symptoms and highlight the importance of comprehensive evaluation in patients with complex presentations. Interestingly, EP is more commonly seen in pediatric populations, making this adult presentation unusual. The nonspecific histopathological findings from the skin biopsy underscore the challenges in diagnosing EP, particularly when sampling is delayed. Despite the lack of specific histological markers, the clinical presentation of acute angiomatous papules with pallor halo is very characteristic of EP. The literature on EP merely reported associated neurological symptoms, making this case unique. It raises important questions about the potential systemic involvement in EP and the need for further research into the pathophysiological connections between dermatological and neurological conditions. This case report aims to enrich the literature with all possible phenomena accompanying this rare dermatological entity. The authors do not claim that EP was related to the patient’s neurological condition in this case. However, it is worth mentioning that vascular responsiveness to altered neurological function might reflect the rash's morphology, i.e., vasodilation areas surrounded by vasoconstricted zonation. More reports are needed for better conclusions. This case report was guided by the CARE checklist protocol.

## Conclusions

This case report highlights the importance of considering EP in the differential diagnosis of sudden-onset erythematous papules encircled by a pale halo. The identification of multiple neurological deficits in this patient underscores the necessity of thorough investigation to uncover potential underlying conditions. This case contributes to understanding EP and its possible systemic implications, emphasizing the need for heightened clinical awareness and further research.
